# Methamphetamine administration increases hepatic CYP1A2 but not CYP3A activity in female guinea pigs

**DOI:** 10.1371/journal.pone.0233010

**Published:** 2020-05-12

**Authors:** Jia Yin Soo, Michael D. Wiese, Rebecca M. Dyson, Clint L. Gray, Andrew N. Clarkson, Janna L. Morrison, Mary J. Berry

**Affiliations:** 1 Early Origins of Adult Health Research Group, University of South Australia, Adelaide, Australia; 2 Health and Biomedical Innovation, University of South Australia, Adelaide, Australia; 3 Department of Paediatrics and Child Health, Brain Health Research Centre and Brain Research New Zealand, University of Otago, Dunedin, New Zealand; 4 Department of Anatomy, Brain Health Research Centre and Brain Research New Zealand, University of Otago, Dunedin, New Zealand; National Institutes of Health, UNITED STATES

## Abstract

Methamphetamine use has increased over the past decade and the first use of methamphetamine is most often when women are of reproductive age. Methamphetamine accumulates in the liver; however, little is known about the effect of methamphetamine use on hepatic drug metabolism. Methamphetamine was administered on 3 occassions to female Dunkin Hartley guinea pigs of reproductive age, mimicking recreational drug use. Low doses of test drugs caffeine and midazolam were administered after the third dose of methamphetamine to assess the functional activity of cytochrome P450 1A2 and 3A, respectively. Real-time quantitative polymerase chain reaction was used to quantify the mRNA expression of factors involved in glucocorticoid signalling, inflammation, oxidative stress and drug transporters. This study showed that methamphetamine administration decreased hepatic CYP1A2 mRNA expression, but increased CYP1A2 enzyme activity. Methamphetamine had no effect on CYP3A enzyme activity. In addition, we found that methamphetamine may also result in changes in glucocorticoid bioavailability, as we found a decrease in 11β-hydroxysteroid dehydrogenase 1 mRNA expression, which converts inactive cortisone into active cortisol. This study has shown that methamphetamine administration has the potential to alter drug metabolism via the CYP1A2 metabolic pathway in female guinea pigs. This may have clinical implications for drug dosing in female methamphetamine users of reproductive age.

## Introduction

Methamphetamine is a potent central nervous system stimulant. At low to moderate doses, it can induce euphoria, altered mental state, tachycardia, anxiety and reduced appetite [1]. Methamphetamine use is a worldwide problem. In 2012, over 100,000 new methamphetamine users were recorded in the United States alone [2]. Similarly, in Australia in 2013–14 there were more than 250,000 regular methamphetamine users [3] whereas New Zealand reported an increase in the percentage of people that have used methamphetamine from 0.2% to 1.8% between 1996–2006 [4]. Coinciding with an increased detection of illegal methamphetamine laboratories [5], there has been an increase in seizures or violations related to methamphetamine and precursor chemicals (pseudoephedrine or ephedrine) [5].

Most studies of the effects of methamphetamine are carried out in males; however, the average age for first methamphetamine use is now ~19 years [2], making it a drug that is widely available to women of child bearing age. Furthermore, the rate of pregnant women presenting to hospital with methamphetamine-related problems has tripled from 8% of total methamphetamine-related presentations in 1994 to 24% in 2006 [6]. The ability of methamphetamine to cross the placenta into the fetal circulation has been shown in human [7] and animal [8] studies. Given that 15–50% of pregnancies are unplanned [9–11], *in utero* methamphetamine exposure is a major public health issue, the full impact of which has not, as yet, been determined.

The direct effects of methamphetamine alone are difficult to assess in humans as ~60% of methampetamine users combine methamphetamine abuse with other drugs [12]. Whilst methamphetamine accumulates in the liver [13, 14], little is known about the effect of methamphetamine on hepatic function, and even less is known about the effect of methamphetamine on hepatic drug metabolism.

Hepatic metabolism usually results in inactivation and detoxification (Phase I) or increased hydrophilicity (Phase II) of drugs. CYPs are a superfamily of hemoproteins that are important enzymes involved in the phase I metabolism of numerous endogenous and exogenous chemicals, including a wide range of illicit (e.g. tetrahydrocannabinol (THC), methamphetamine and ecstasy [15, 16]) and therapeutic drugs. CYP3A is probably the most important CYP with regard to drug metabolism, as it metabolises approximately 30% of exogenous chemicals, and CYP1A2 plays a significant role in the metabolism of several medicines, including analgesics and antipyretics (paracetamol, lignocaine), antipsychotics (olanzapine, clozapine) and antidepressants [17]. Hepatic drug transporters such as P-glycoprotein (P-gp) and breast cancer resistant protein (BCRP) also play an important role in the regulation of drug concentrations in the hepatocyte [18]. Methamphetamine increases activity of CYP3A in male rats [19]; however, the effects of methamphetamine on female cytochrome P450 enzyme activity is not known. Drug metabolism profiles are sex-specific [20–23] and thus it is essential to understand the full impact of methamphetamine use in females, particularly those of reproductive age. However, there is a paucity of data examining the impact of methamphetamine and other drugs in females, especially those of reproductive age, limiting the clinicians ability to predict the maternal and/or fetal consequences of drug exposure.

**Fig 1:**
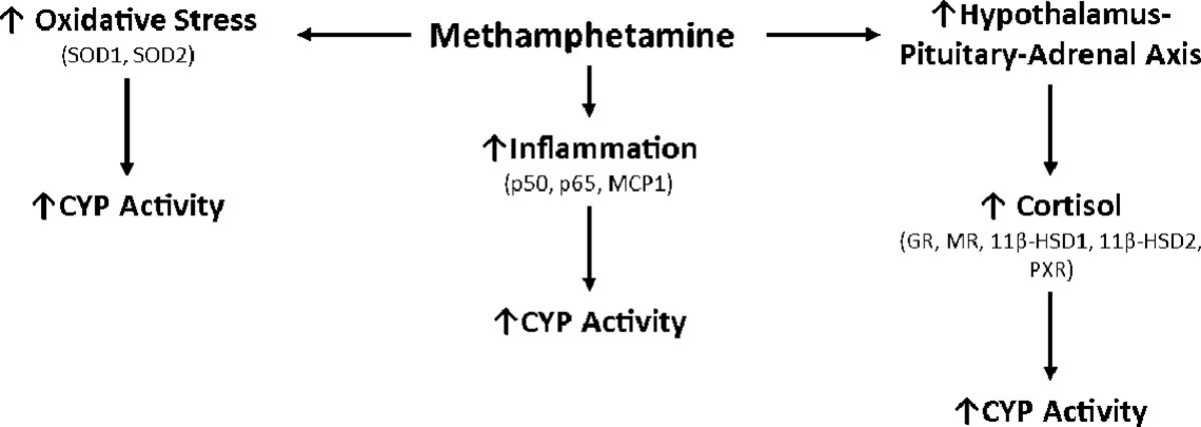
Overview of pathways that can be dysregulated by methamphetamine. GR: glucocorticoid receptor, MR: mineralocorticoid receptor, 11β-HSD: 11β-hydroxysteroid dehydrogenase types 1 and 2, PXR: pregnane X receptor, MCP-1: Monocyte Chemoattractant Protein-1, SOD: Superoxide dismutase types 1 and 2, CYP: Cytochrome P450.

Methamphetamine has been shown to dysregulate glucocorticoid [24], inflammatory [25] and oxidative stress [26] pathways, and in turn these pathways can modify CYP activity (Fig 1). For example, CYP function can be influenced by a range of transcription factors such as pregnane X receptor (*PXR*), which upregulate CYP3A in the presence of toxic substances and steroids [27, 28].

**Fig 2:**
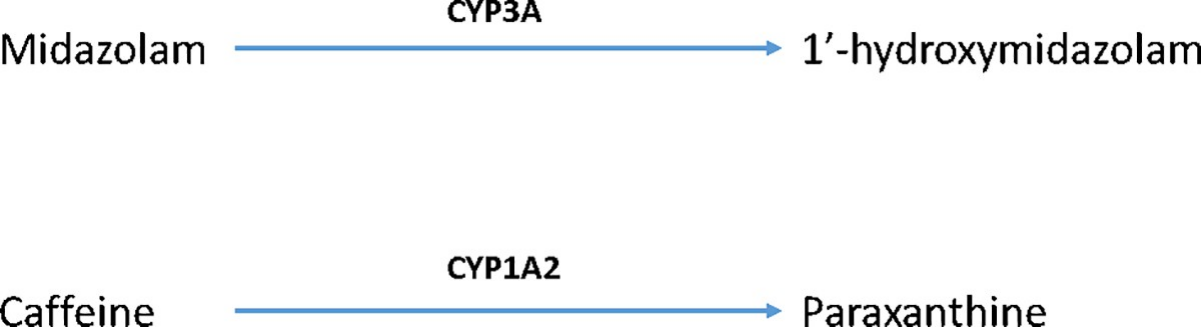
CYP probes. Specific reactions used to determine CYP activity in the present study.

Functional activity of CYPs *in vivo* can be determined by administering a low dose of a probe drug that is metabolised by a specific CYP to a known metabolite and measuring the concentration of the parent drug and its metabolite over time (Fig 2). Enzyme activity is determined by calculating the exposure of the metabolite produced (as measured by the Area Under the Concentration-time curve, AUC) relative to the exposure to parent drug. For studies in humans where CYP activity is assessed, this is a common approach [29, 30].

As such, this study aimed to determine the effect of methamphetamine exposure in female guinea pigs of reproductive age on different factors that can regulate CYP450 activity and enzyme activity of CYP1A2 and CYP3A.

## Methods and materials

### Materials

Acetonitrile (Honeywell Australia, North Ryde, Australia); caffeine citrate (Biomed Limited, Auckland, New Zealand); internal standard caffeine d3 (Toronto Research Chemicals, Toronto, Canada); Formic acid (Sigma Aldrich, New South Wales, Australia); isoflurane (Attane^TM^, Bayer, Auckland, New Zealand); KiCqStart SYBR Green qPCR ReadyMix Low Rox (Sigma Aldrich, New South Wales, Australia); methamphetamine (BDG Synthesis, Wellington, New Zealand); internal standard methamphetamine d5 (Toronto Research Chemicals, Toronto, Canada); midazolam (Claris, AFT Pharmaceutical Limited, Auckland, New Zealand); internal standard midazolam d6 (Toronto Research Chemicals, Toronto, Canada); internal standard paraxanthine d3 (Toronto Research Chemicals, Toronto, Canada); QIAZOL Lysis Reagent (QIAGEN, Victoria); RNeasy Mini Kit (QIAGEN, Victoria); sodium pentobarbital (Pentobarb, Provet, Palmerston North, New Zealand).

### Ethical approvals and animals

All procedures were approved by the University of Otago, Wellington Animal Ethics Committee. Eight sexually mature female virgin Dunkin Hartley guinea pigs (33–49 weeks old; breeding colony in the Biomedical Research Unit at University of Otago, Wellington) were allocated into either saline (Control; n = 4) or methamphetamine (MA; n = 4) treatment groups, randomly matched for age and weight at commencement of study [31]. Guinea pigs were housed individually in a 12:12 hr light cycle. Methamphetamine (5 mg/kg) or an equivalent volume of saline was administered subcutaneously on three alternate days to mimic recreational drug exposure.

### Functional analysis of drug metabolism

One week before the first methamphetamine exposure and 2 hours after the third methamphetamine exposure, test drugs containing low dose of midazolam (0.03 mg/kg) and caffeine (5 mg/kg) was administered orally to the MA group. Blood samples (300 μl) were collected from the ear vein at 1, 2, 3, and 4 hrs after its administration. Each animal therefore acted as its own control for the effect of methamphetamine on hepatic drug metabolism.

### Post mortem and tissue collection

Eight hours after the third methamphetamine dose, anaesthesia was induced by chamber inhalation of 4% isoflurane and maintained by mask inhalation of 2–2.5% isoflurane in 100% O_2_ (BOC Gas, New Zealand). Once a deep surgical anaesthesia was obtained, the liver was excised, weighed, snap frozen in liquid nitrogen and stored at -80°C until analysis. Blood was collected by cardiac puncture into EDTA vacutainers. Blood samples were spun at 4000*g* for 10 minutes and plasma was collected and stored at -80°C until analysis. Guinea pigs were then euthanised with an intracardiac overdose of sodium pentobarbital (>100 mg/kg).

### Methamphetamine assay

Plasma concentrations of methamphetamine were determined via liquid chromatography-tandem mass spectrometry (LC-MS/MS) as previously described with slight modifications [32]. Plasma (50 μL) was diluted with 50 μL MilliQ water, internal standard (methamphetamine-d5) was added and plasma protein was precipitated via the addition of acetonitrile (100 μL) and 0.2M zinc sulfate (100 μL). 10 μL was injected onto a Titan C18 UHPLC column (1.9 μM, 2.1X100 mm, Sigma Aldrich, New South Wales, Australia). Mobile phase A was 0.05% formic acid in water and mobile phase B was acetonitrile. The flow rate was 0.3 mL/min, and Mobile phase B was run at 10% for 1 minute, and then increased to 95% over 4 minutes. This was held for 1 minute, and then returned to 10% for 2 minutes until the next injection. Methamphetamine was detected using a Shimadzu 8060 LC-MS/MS (Shimadzu Kyoto, Japan), system in positive ion mode by determination of the area obtained from the methamphetamine peak to that of the internal standard (d5-methamphetamine), and comparing to a standard curve where known concentrations of methamphetamine (5–500 ng/mL) were added to blank plasma.

### Measurement of functional activity of CYP1A2 and CYP3A4 enzymes

Plasma (100 μL) was precipated using 900 μL of acetonitrile and 50μL of internal standard mastermix (10 ng/mL midazolam d6, 500 ng/mL caffeine d3 and 1500ng/mL paraxanthine d3) was added. The mixture was vortexed and centrifuged at 10,000 *g* for 10 minutes at 4°C. The clear supernatant was collected and evaporated to dryness using a Concentrator plus (Eppendorf). The samples were reconstituted using 200 μL of 20% acetonitrile in water. Analytes were separated using a reverse phase liquid chromatography (LC) method whereby 30 μL of sample was injected onto a Kinetex EVO 2.6 μM, 2.1X100 mm, C18 LC column (Phenomenex, Torrance, CA). Mobile phase A was 0.05% formic acid in water and mobile phase B was 100% acetonitrile. The flow rate was 0.3 mL/min, and mobile phase B was initially 20%, which was increased linearly to 100% over 4 minutes. Analytes were detected using LC-MS/MS (LCMS8060, Shimadzu, Kyoto, Japan), which was operated in positive ion mode. The area fo each analyte was compared to the area of the relevant internal standard (midazolam-d6 was used for hydroxymidazolam) and concentration in each sample was calculated using standard curves prepared in blank plasma in the range of 1–200 ng/mL (midazolam and hydroxymidazolam) and 25–5000 ng/mL (caffeine and paraxanthine).

The area under the curve (AUC) of caffeine, paraxanthine, midazolam and hydroxymidazolam between administration and 6 hours was determined via non-compartmental analysis using PK Solver [33], and the metabolic activity of CYP1A2 and CYP3A were determined as the ratio between the AUC of caffeine:paraxanthine and midazolam:hydroxymidazolam respectively.

### Quantification of gene expression

#### Total RNA extraction

To ensure all essential steps in the protocol were included, we followed the MIQE guidelines [34]. Total RNA extraction (Control, n = 4; MA, n = 4) was carried out using QIAZOL Lysis Reagent and RNeasy Mini Kit according to the manufacturer’s instructions as previously described [35, 36]. Total RNA was quantified by spectrophotometric measurements at 260 and 280 nm using NanoDrop^™^ Lite Spectrophotometer (Thermo Fisher Scientific, Australia). Protein and DNA contamination in each sample was checked using 260/280 nm ratio results. RNA integrity was assessed by verifying the RNA bands on a 1% agarose gel. cDNA was synthesised as previously described [35]. Genomic DNA and reagent contamination were tested using controls containing either no Superscript III (No Amplification Control (NAC)) or no RNA transcript (No Template Control (NTC)), respectively.

#### Primer design and validation

Primer pairs for breast cancer resistant protein (*BCRP*), cytochrome P450 1A2 (*CYP1A2*), pregnane X receptor (*PXR*), glucocorticoid receptor (*GR*), 11β-hydroxysteroid dehydrogenase (*11βHSD*) 1, *11βHSD2*, mineralocorticoid receptor (*MR*), *p50*, *p65*, monocyte chemoattractant Protein (*MCP*) *1*, Superoxide dismutase (*SOD*) *1* and *SOD2* were designed using Cavia porcellus sequences. Primer concentrations were optimised (S1 Table), validated to generate a single transcript and the product was sequenced by Australian Genome Research Facility Ltd.

#### Quantitative real-time PCR

*β actin*, *beta-2 microglobulin* (*B2M*) and *18S* were selected from a panel of reference genes using the geNorm component of the qBase 2.0 relative quantification model (Biogazelle, Belgium) as they were expressed stably across treatment groups (Control vs. MA) [36–38].

The mRNA expression of P-glycoprotein (*P-gp*) [36], *CYP1A2*, *PXR*, *GR*, *11βHSD1*, *11βHSD2*, *MR*, *p50*, *p65*, *MCP1*, *SOD1*, *SOD2* and reference genes in liver samples were measured using KiCqStart SYBR Green qPCR ReadyMix Low Rox on a ViiA7 Fast Real-time PCR system (Applied Biosystems, California, USA) as previously described [35, 36]. The reactions were quantified by setting the threshold within the exponential growth phase of the amplification curve and obtaining corresponding C_t_ values. DataAssist Software v3.0 (Applied Biosystems) [38] was used to find the 2^−ΔCt^, which shows the abundance of each transcript relative to the abundance of the three stable reference genes and is expressed as mean normalized expression. DataAssist 3.0 analysis software (Applied Biosystems, California, USA) was used to normalise the abundance of target genes relative to the abundance of reference genes and expressed as mRNA mean normalised expression (MNE) ± SEM.

### Statistical analysis

Body and liver weight and gene expression data are presented as mean ± standard error of the mean (SEM) and analysed using unpaired Student’s t-test. Enzyme function data taken pre and post methamphetamine administration was analysed using paired t-test. A probability level of 5% (*P*<0.05) was considered statistically significant.

## Results

### Physical characteristics of guinea pigs

There was no difference in body weight at the beginning of the protocol (Control: 872 ± 57g; MA: 888 ± 61g) or at post-mortem (Control: 867 ± 55g; MA: 854 ± 54g) between Control and MA groups. There was also no difference in whole liver weight (Control: 27.3 ± 1.3g; MA: 26.3 ± 1.1g) or liver weight when expressed relative to body weight (Control: 3.2 ± 0.1g/g; MA: 3.1 ± 0.2g/g) between the Control and MA groups. There were no methamphetamine-related deaths during this study.

### Methamphetamine plasma concentration

**Fig 3:**
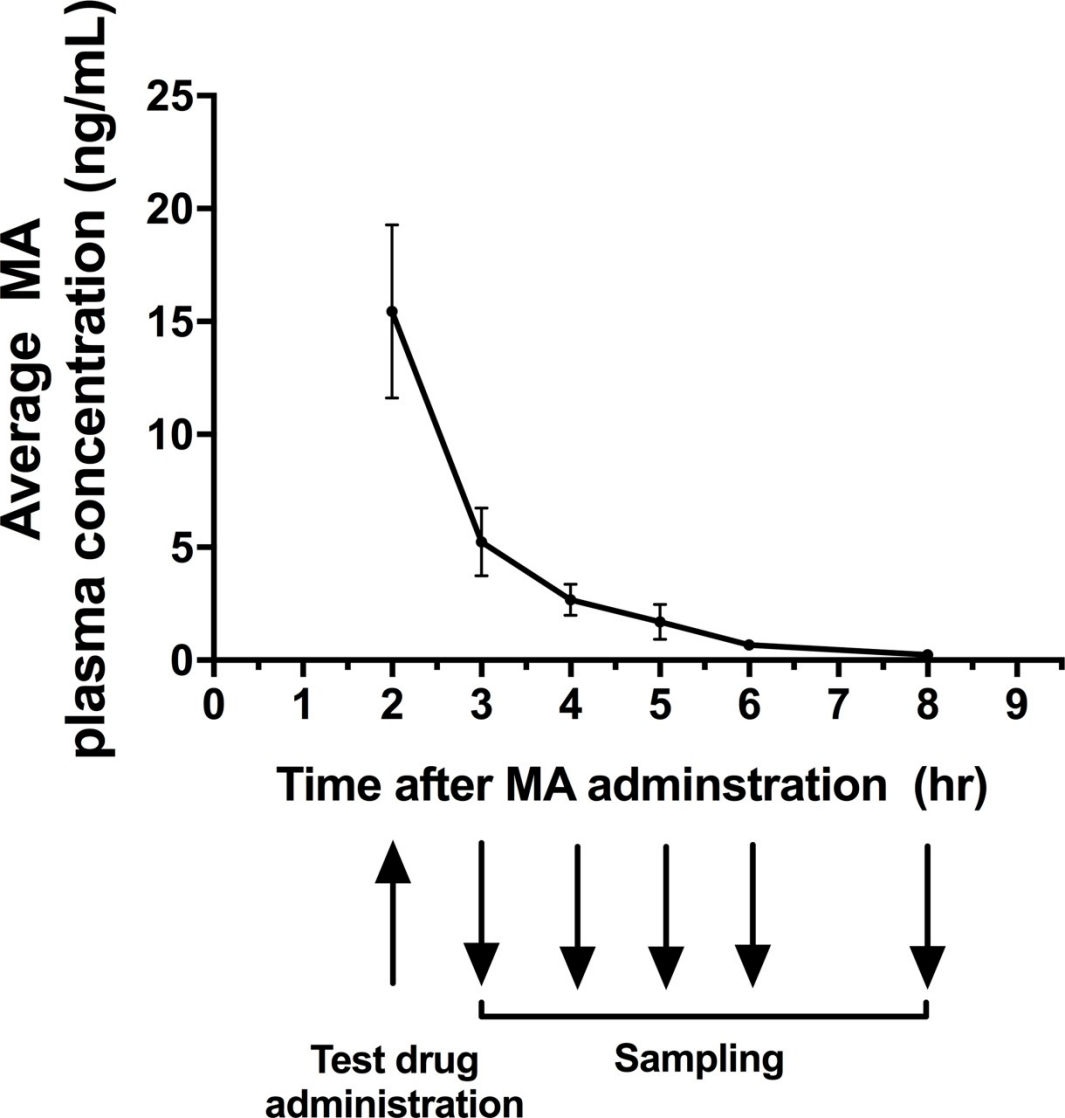
Methamphetamine plasma concentration (n = 4) vs time (hours) after third methamphetamine dose. Test drugs caffeine (metabolised by CYP1A2) and midazolam (metabolised by CYP3A) was administered 2 hours after the methamphetamine dose and plasma samples were collected 3, 4, 5, 6 and 8 hours after methamphetamine dose. Error bars represent standard error of mean (SEM).

The mean maximum plasma concentration of methamphetamine (first blood sample taken 2 hours after administration) was 15.5 ± 7.7 mcg/mL. The plasma concentration of methamphetamine dropped rapidly in the first four hours after administration (Fig 3). The average area under the curve (AUC) was 27 ± 6.9 mcg mL^-1^ hr.

### Effect of methamphetamine administration on hepatic cytochrome P450 enzymes

**Fig 4:**
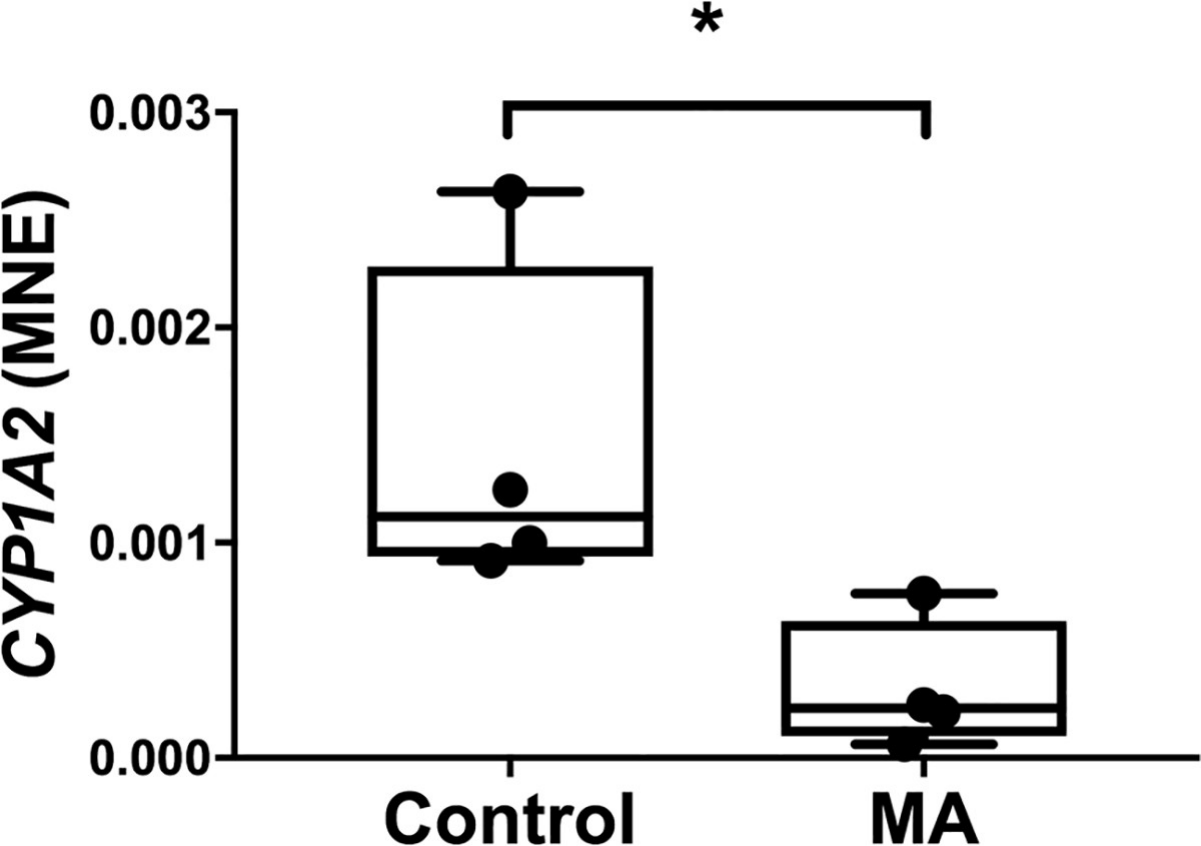
Normalised mRNA expression of hepatic drug metabolising enzyme *CYP1A2* (A) between Control (n = 4) and MA (n = 4) groups. MNE, mean normalised expression; MA, methamphetamine; unpaired t test; *P<0.05 from Control.

**Fig 5:**
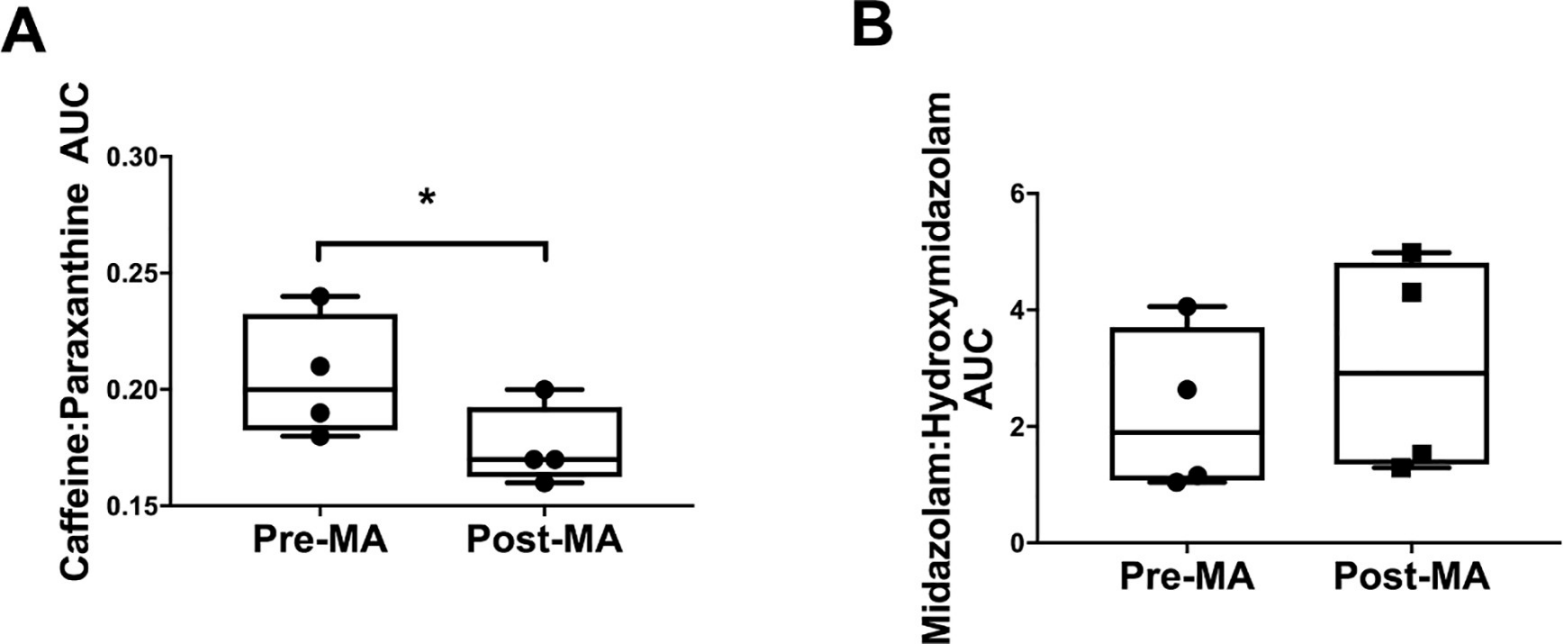
Functional activity of hepatic CYP1A2 (A) and CYP3A (B) before (Pre-MA) and after (Post-MA) methamphetamine exposure (n = 4). MA, methamphetamine; AUC, area under the curve; paired t test; *P<0.05 from Pre-MA.

Methamphetamine administration reduced the mRNA expression of *CYP1A2* compared to controls (MA; 0.0032 ± 0.0002 vs 0.00105 ± 0.0001, *P* = 0.014, Fig 4). In the 4 animals administered MA, the caffeine:paraxanthine AUC was reduced after MA administration (pre vs post MA 0.205 ± 0.013 vs 0.175 ± 0.009, *P* = 0.0138, Fig 5A), suggesting increased activity of CYP1A2.

No significant effect of MA administration on the functional activity of the CYP3A enzyme was apparent (pre vs post MA midazolam:hydroxymidazolam AUC 2.22 ± 0.71 vs 3.02 ± 0.945, *P* = 0.095, Fig 5B).

### Effect of methamphetamine on hepatic drug transporter gene expression

**Fig 6:**
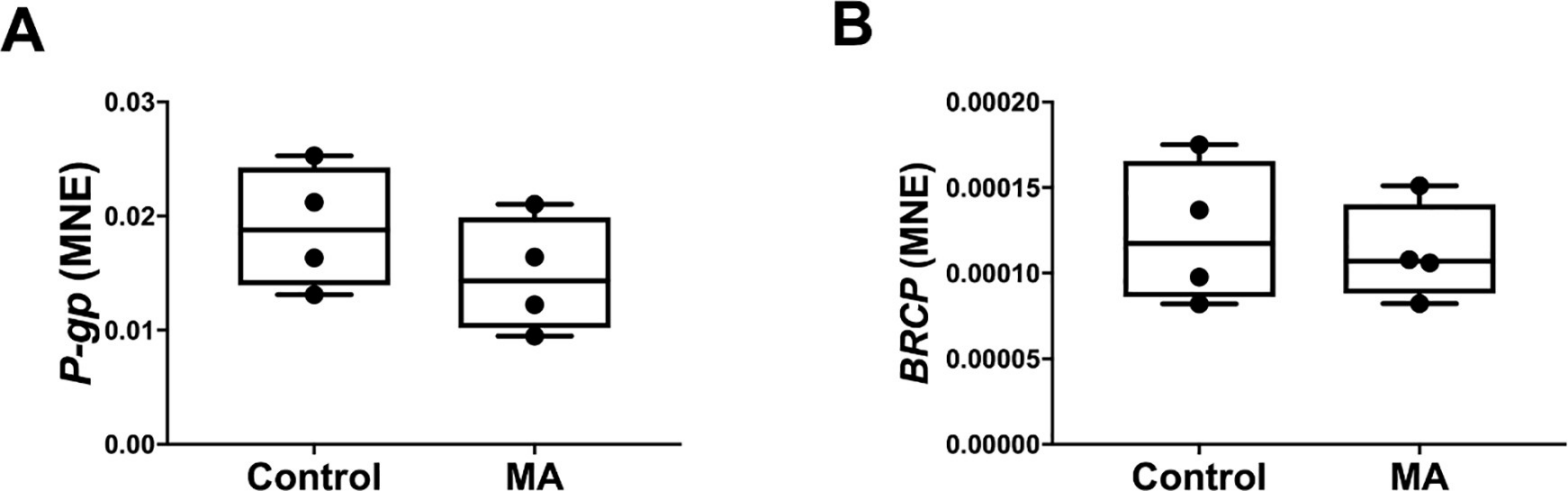
Normalised mRNA expression of hepatic drug transporters *P-gp* (A) and *BCRP* (B) between Control (n = 4) and MA (n = 4) groups. MNE, mean normalised expression; MA, methamphetamine; unpaired t test; *P<0.05 from Control.

Methamphetamine administration had no effect on hepatic mRNA expression of the drug *P-gp* (Control: 0.019 ± 0.003 vs MA: 0.015 ± 0.002, *P* = 0.297 Fig 6A) and *BCRP* (Control: 0.00012 ± 0.00002 vs MA: 0.00011 ± 0.00001, *P* = 0.673, Fig 6B).

### Effect of methamphetamine on hepatic glucocorticoid bioavailability and regulator of drug transporter and drug metabolising enzyme

**Fig 7:**
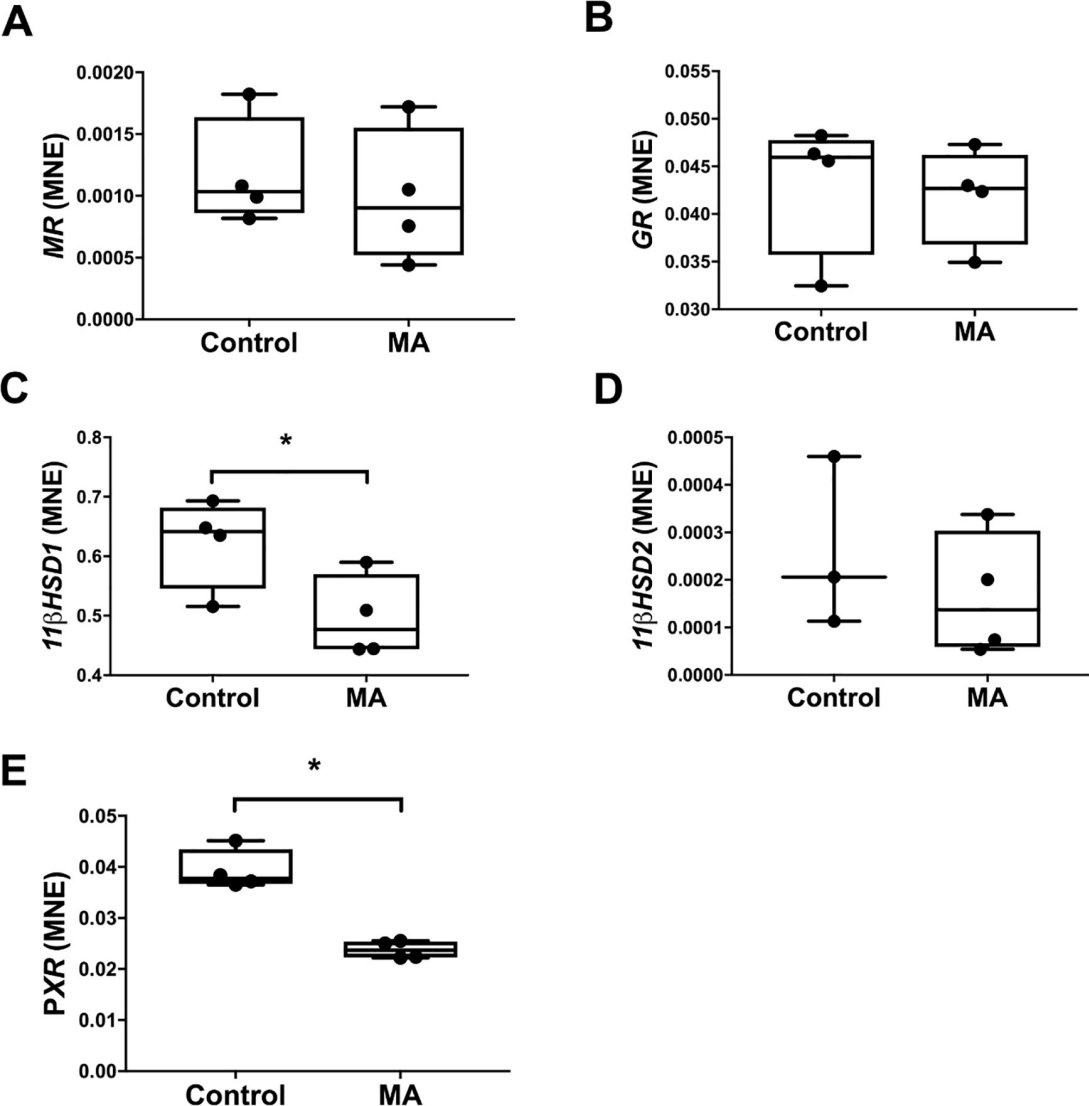
Normalised mRNA expression of hepatic regulators of drug transporter and metabolising enzyme regulators *MR* (A), *GR* (B), *11βHSD1* (C), *11βHSD2* (D) and *PXR* (E) between Control (n = 4) and MA (n = 4) groups. MNE, mean normalised expression; MA, methamphetamine; unpaired t-test; *P<0.05 from Control.

Methamphetamine administration had no effect on mRNA expression of *GR*, *MR* or *11βHSD2*, a glucocorticoid inactivating enzyme (Fig 7A, 7B & 7D).Methamphetamine administration resulted in a decrease in hepatic mRNA expression of *11βHSD1* (Control: 0.623 ± 0.038 vs MA: 0.497 ± 0.035, *P* = 0.049, Fig 7C) and *PXR* (Control: 0.039 ± 0.002 vs MA: 0.024 ± 0.001, *P*<0.001, Fig 7E).

### Effect of methamphetamine on hepatic inflammatory markers

**Fig 8:**
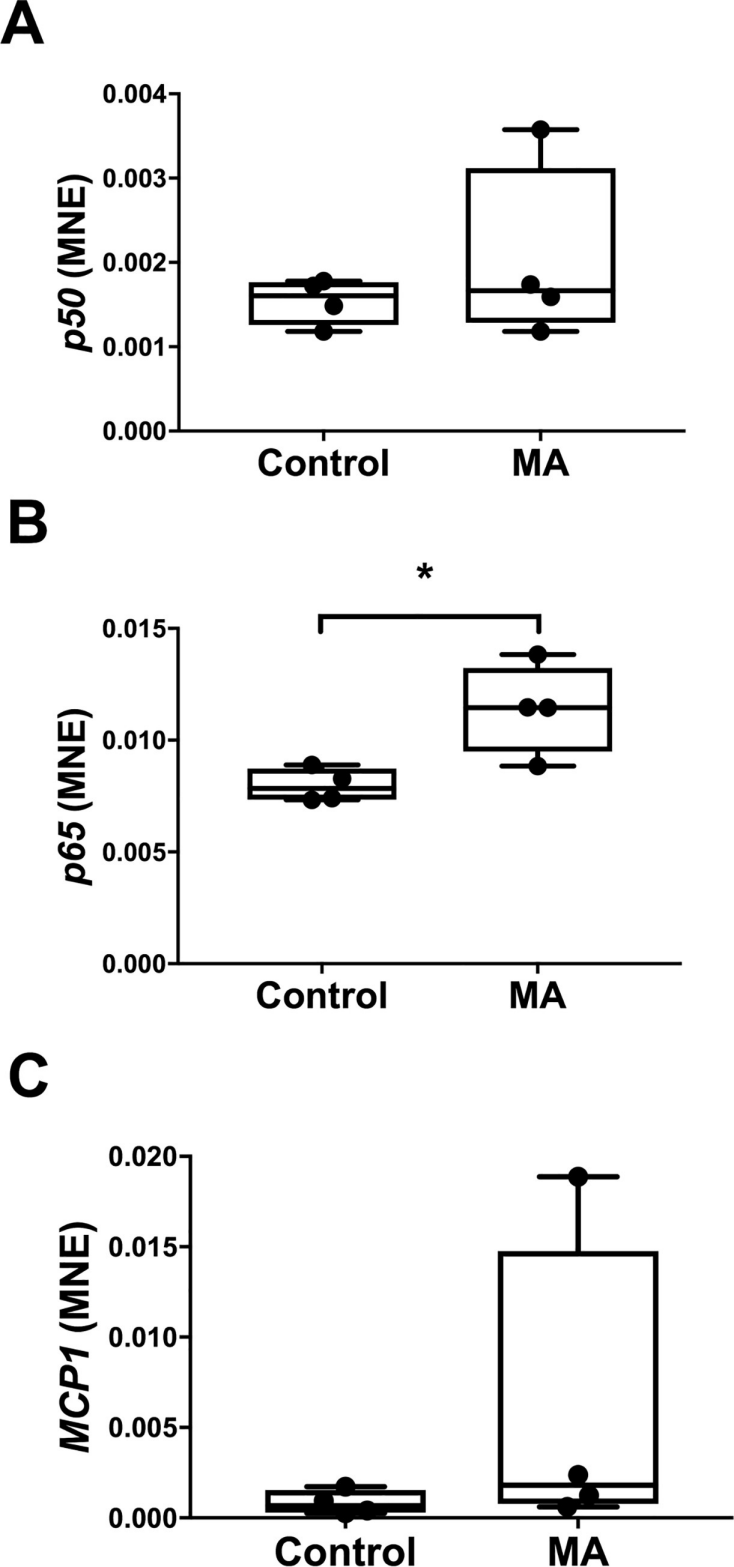
Normalised mRNA expression of hepatic markers of inflammation *p50* (A), *p65* (B) and *MCP1* (C) between Control (n = 4) and MA (n = 4) groups. MNE, mean normalised expression; MA, methamphetamine; unpaired t test; *P<0.05 from Control.

Hepatic gene expression of *p65* was increased in animals who received MA (Control: 0.008 ± 0.0004 vs MA: 0.0114 ± 0.001, *P* = 0.02, Fig 8B). However, no significant effect on hepatic gene expression of *p50* or *MCP1* was apparent in those animals who received MA compared to controls (Fig 8).

### Effect of methamphetamine on hepatic antioxidant enzyme

**Fig 9:**
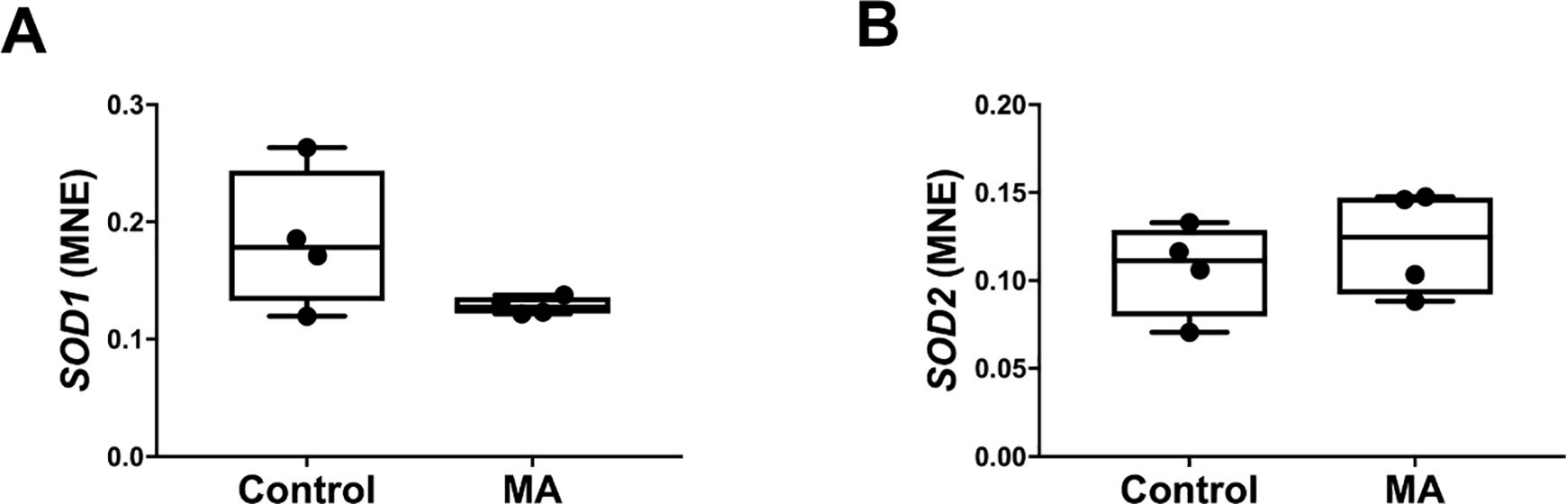
Normalised mRNA expression of hepatic *SOD1* (A) and *SOD2* (B) between Control (n = 4) and MA (n = 4) groups. MNE, mean normalised expression; MA, methamphetamine; unpaired t test; *P<0.05 from Control.

Methamphetamine administration had no effect on hepatic gene expression of *SOD1* (Control: 0.185 ± 0.03 vs MA: 0.128 ± 0.004, *P* = 0.108) or *SOD2* (Control: 0.107 ± 0.013 vs MA: 0.121 ± 0.015, *P* = 0.488, Fig 9).

## Discussion

This study shows that methamphetamine exposure can change hepatic drug metabolism in females of reproductive age, a population that is often underrepresented in both human and animal studies [39]. Dose and frequency of methamphetamine use varies widely amongst users and is therefore difficult to estimate. Self-reported doses range from 50–500 mg and may be up to 4 g per day [40], although since illicit methamphetamine in society is unlikely to be pure, it is often difficult to assess the doses commonly used in humans, even if studies have reported the doses used. As guinea pigs do not tolerate high initial doses of methamphetamine (e.g. more than 6 mg/kg [41]), we chose to give a dose of 5 mg/kg in female guinea pigs of reproductive age. In order to ensure that we have ethically conducted this study with minimal mortality, the dose of methamphetamine was selected based on the pilot studies testing dose tolerability in guinea pigs. Higher doses caused negative outcomes and was therefore discontinued. Using a dose of 5mg/kg, we measured an average plasma concentration of at least 15.5 mcg/L (~0.016 mg/L), which is below concentrations found in methamphetamine-related deaths (0.02–15.0 mg/L) [42].

Herein, we examined three different pathways that can be dysregulated by methamphetamine: glucocorticoids, inflammation and oxidative stress. Glucocorticoids can regulate CYP450 through direct binding of glucocorticoid receptor (GR) to glucocorticoid responsive element, complexing with other transcription factors to activate promoter DNA or by regulating other CYP450 regulators such as pregnane X receptor (PXR) [43]. Glucocorticoids increase the expression of CYP3A [44]. Methamaphetamine can activate the hypothalamic-pituitary-adrenal axis [45], with chronic methamphetamine administration in male rats increasing the expression of *GR* in the hippocampus [46]. Although we did not find any changes to *GR* in the liver of female guinea pigs, we showed that methamphetamine exposure decreases the mRNA expression of *11βHSD1*, an enzyme that converts inactive cortisone to active cortisol, suggesting that there may be a decrease in glucocorticoid bioavailability in the liver.

Methamphetamine exposure increases nuclear factor kappa-light-chain-enhancer of activated B cells (NF-κB) binding to DNA and this binding resulted in reduced superoxide dismutase (SOD) 1 in transgenic mice [47]. NF-κB, comprising of p65 and p50, is a key regulator of immunity and inflammation [48]. Unlike the present study, methamphetamine exposure in male mice increases plasma MCP1 concentrations [49], which may be due to significantly longer methamphetamine exposure in those studies compared to the current study. However, we found that methamphetamine exposure increased p65 mRNA expression, a subunit of the NF-κB, in the liver of female guinea pigs, suggesting that even short duration methamphetamine exposure may increase local hepatic inflammation in females.

In this study, we found no changes in *SOD 1* or *2* mRNA expression; members of a family of enzymes that play an important role in protecting against reactive oxygen species and superoxide anion radicals. This is in line with previous studies that found short duration methamphetamine exposure, similar to that used in the present study, did not affect hepatic SOD expression in male mice. However, chronic methamphetamine exposure for one month in male mice increased cardiac *SOD2* mRNA expression, suggesting an increase in oxidative stress [50]. In SOD knockout/CYP3A, overexpression hepatocytes, treatment with known hepatotoxic drugs causes an increase in superoxide production and reduces cell viability [51]. In addition, male SOD2 knock-out mice exposed to paracetamol have an an increased risk of hepatotoxictiy [52], suggesting that SOD2 may be important in protecting the liver from oxidative stress during drug metabolism.

Cytochrome P450 enzyme activity can be measured through *ex vivo* methods, such as microsomes and isolated perfused liver, and *in vivo* methods, such as the Geneva cocktail [53]. In this study, we chose to use an *in vivo* method of measuring CYP1A2 and CYP3A activity using a “cocktail” method [53]. This *in vivo* approach accounts for variability in both gene expression and enzyme activity, which can be influenced by underlying differences in enzyme function due to genetic polymorphisms, and post-translational effects such as enzyme inhibition due to drugs, hormones or microRNA.

We found methamphetamine exposure increases CYP1A2 enzyme activity despite a decrease in *CYP1A2* mRNA expression. CYP1A2 is responsible for the metabolism of a variety of drugs that are used in women of childbearing age or pregnant women such as analgesics (paracetamol), antidepressants (fluoxetine and duloxetine), antiemetics (ondansetron) and cardiovascular regulators (propranolol) [54]. The expression of the CYP1A family of enzymes can be regulated by glucocorticoids, although multiple mechanisms are thought to be involved, many of which remain to be elucidated [55]. For example, hepatic CYP1A2 mRNA expression can be induced by dexamethasone, a synthetic glucocorticoid, although in the same study CYP1A2 activity was reduced [55]. In the present study, methamphetamine administration decreased hepatic mRNA expression of *11βHSD1*, which may have reduced the conversion of cortisone to active cortisol, subsequently decreasing *CYP1A2* mRNA expression. We found an increase in CYP1A2 activity in MA-exposed animals and this may be mediated by the suggested reduction in active hepatic glucocorticoid bioavailability in the methamphetamine exposed female guinea pig [55]. However, we have not fully elucidated the paradoxical reduction in *CYP1A2* mRNA expression and increase in enzyme activity. In addition, we found a decrease in *PXR* expression in the methamphetamine exposed animals. The regulation of *PXR* expression is not fully understood. However, there is evidence that glucocorticoids can induce the expression of PXR in human hepatocytes [56]. The reduction in *PXR* expression may also be due to less cortisol in the methamphetamine exposed compared to the control guinea pigs. Hence, despite the small sample size, this study provides preliminary evidence that methamphetamine exposure in females of childbearing age may reduce the effectiveness of drugs metabolised by CYP1A2 due to increased hepatic metabolism. such as ondansetron and paracetamol [54].

Pre-treatment with methamphetamine increased the metabolism of midazolam in male rats [19]. However, in the present study CYP3A activity was unaffected in female guinea pigs exposed to methamphetamine. This may be due to several factors, including species variation and sex. Sex differences in drug metabolism are well documented, especially the expression and activity of CYP3A [21, 22]. Unfortunately, we were not able to determine CYP3A expression in guinea pigs, as there are potentially 3 different CYP3A enzymes (CYP3A14, 3A15, and 3A17) in guinea pigs, and primers were not available for these enzymes. Females have higher protein expression and activity of CYP3A compared to males [23], the difference in both protein expression and activity of CYP3A may reflect the difference in regulation of CYP3A in males and females, and thus explain the different result in this study. In addition, although male methamphetamine treated mice had lower concentration of dopamine in the brain compared to female methamphetamine treated mice, methamphetamine treatment resulted in a decrease in striatal dopamine in both males and females, which may indicate that the effects of methamphetamine manifests differently in males and females [57].

Although sex differences in drug response and metabolism are well documented, females continue to remain underrepresented in clinical trials [20] and overall, in both animal and human studies, females are underrepresented [39], especially females of childbearing age. In addition, as many as 50% of all pregnancies worldwide are unplanned; thus, the potential effect of illicit and prescribed drug exposures on the health of the offspring remains to be understood. There is therefore an urgent unmet need to understand how drugs affect females, especially those of reproductive age as there are profound implications for health and wellbeing that may extend into the next generation.

A relative limitation of the study is the small sample size. However, it should be noted that with this sample size there was a decrease in CYP1A2 gene expression and functional activty after exposure to methamphetamine. We also showed that methamphetamine changed the gene epxression of molecules known to regulate CYP expression and function. It is possible that with a larger sample size observed trends in functional activity of CYP3A following methamphetamine exposure would have become more pronounced; however, as power analysis suggests that this would have required at least doubling the sample size we choose to use the minimum number of animals to establish the proof of concept described above. Finally, humans self-administer methamphetamine but may have medical conditions requiring other therapeutic medications. Greater understanding of the impact of methamphetamine on the metabolism of other medications is required to support better health outcomes for those with methamphetamione addiction and medical co-morbodities.

## Conclusion

This study has shown that three doses of methamphetamine over a relatively short period may be sufficient to increase drug metabolism by CYP1A2 in females of reproductive age. This is of concern as the change in drug metabolism due to methamphetamine exposure may affect the clearence or bioactivation of other drugs that are consumed simultaneously with methamphetamine or drugs that are used to treat mother or baby, and ultimately affect either efficacy and/or toxicity of drugs that are metabolised through the CYP1A2 pathway.

## Supporting information

S1 Table Primer sequence, concentration and accession number. (DOCX)

S1 Data (XLSX)
