# Supplementary material for: Methamphetamine administration increases hepatic CYP1A2 but not CYP3A activity in female guinea pigs
Source: PLoS One. 2020 May 12;15(5):e0233010. doi: 10.1371/journal.pone.0233010 (PMC7217439; doi:10.1371/journal.pone.0233010)
Supplement: S1 Table — (DOCX) [file pone.0233010.s001.docx]

**Supplementary Table 1**. Primer sequence, concentration and accession number.

| **Primer Name** | **Sequence 5’ 🡪 3’** | **Primer Conc.**  **(nM)** | **Accession**  **No.** |
| --- | --- | --- | --- |
| ***Drug transporter and metabolising enzyme*** | | | |
| *BCRP*  *Forward*  *Reverse* | AGTTCTCAGCAGCTCTTCGGCT  CCCCAGACACACCACGGATAAAC | 50  50 | NM_011920.3 |
| *CYP1A2*  *Forward*  *Reverse* | GAAGCCAGAGATCCAGAAGAAG  AGGGCAGGAAGGAAGAGTAT | 450  450 | D50457.1 |
| ***Regulators of drug transports and metabolising enzymes*** | | | |
| *PXR*  *Forward*  *Reverse* | CGGGCCATGAAACGCAATGTCC  TTCTTCATGCCGCTCTCCAGGC | 900  900 | AF151377 |
| *GR*  *Forward*  *Reverse* | AGACCTGTTGATGGATGAAA  TTTTAGGTTTAGTGTCCGGT | 450  450 | L13196 |
| *11βHSD1*  *Forward*  *Reverse* | TTGTCACAGGGGCCAGCAAAGG  CCAAGTTCTAAGCAGCGGGCCA | 450  450 | AF188005.1 |
| *11βHSD2*  *Forward*  *Reverse* | CTTCATTAGTCCCTGTCTGC  TAGGTTTGAGTTCCCTCG | 450  450 | AF411014.1 |
| ***Regulators of oxidative stress*** | | | |
| *SOD1*  *Forward*  *Reverse* | AGGACTCTCTGATCTCACTCTC  TCCAGCATTTCCCGTCTTT | 900  900 | XM_003467249.2 |
| *SOD2*  *Forward*  *Reverse* | CCCGATTTACCCTACGACTATG  GTACTTCTCCTCAGCGATGTT | 900  900 | XM_003466367.2 |
| ***Regulators of inflammation*** | | | |
| *p50*  *Forward*  *Reverse* | AAAAGGAGCTCATCCGCCAGGC  AACATGAGCCGCACCACGCT | 450  900 | ENSCPOT00000007  863 |
| *p65*  *Forward*  *Reverse* | TACGACCTGAATGCTGTGCGGC  GGCGCGGTTGTCAAAGATGGGA | 450  450 | ENSCPOT00000025  259 |
| *MCP1*  *Forward*  *Reverse* | GCTTGTGCTCCAACACTCCA  ACCCACTTCTGTGTGGGGTC | 450  450 | NM_001172926.1 |
